# Ecological, genetic and geographical divergence explain differences in colouration among sunbird species (Nectariniidae)

**DOI:** 10.1002/ece3.11427

**Published:** 2024-09-11

**Authors:** M. P. J. Nicolaï, S. Rogalla, M. Yousefi, R. C. K. Bowie, L. D'Alba, M. D. Shawkey

**Affiliations:** ^1^ UGent Gent Belgium; ^2^ Biofisika Institute Leioa Spain; ^3^ Damghan University Damghan Iran; ^4^ Leibniz Institute for the Analysis of Biodiversity Change (LIB), Museum Koenig Bonn Germany; ^5^ Museum of Vertebrate Zoology and Department of Integrative Biology University of California Berkeley California USA; ^6^ Naturalis Biodiversity Center Leiden the Netherlands

**Keywords:** light environment, speciation, species isolation

## Abstract

How extravagant ornamental traits evolve is a key question in evolutionary biology. Bird plumages are among the most elaborate ornaments, displaying almost all colours of the rainbow. Why and how birds evolved to be so colourful remains an open question with multiple and sometimes competing hypotheses. Different colours in different patches (i.e. body parts) might have different functions and thus result from different forms of selection (e.g. natural vs. sexual selection). Here we test the influence of three factors on colour diversity in sunbirds: (1) geographical distance, (2) differences in light environment and (3) phylogenetic distances. We show that both natural and sexual selection affect the evolution of sunbird colouration, but that their extent and direction differs between sexes, and varies with the extent of species overlap and across different patches on the body. Even though overlap in light environment partially explains colour differences among species, no colour metric (brightness, hue or chroma) covaries with light environment. Our results suggest that multiple forms of selection influence the colouration of different colour patches in different ways across an organism's body, highlighting the need to investigate colouration as a network of individual but inter‐connected colour patches. These results are likely to be generalizable across the multitude of colourful animals.

## Graphical abstract

Sunbird colour diversity is explained by genetic, geographical and ecological differences. These different mechanisms have different effects on different parts of the body. This suggests that multiple forms of selection influence colouration of animals.

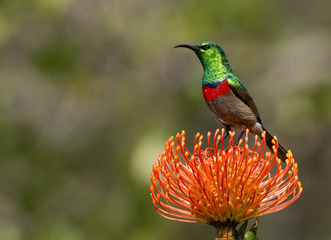

## 1 INTRODUCTION

Animal ornamentation, from the lion's mane to the peacock's tail, has caught the attention of biologists for centuries (Darwin, 1871). One of the most common and widespread forms of ornamentation is the bright colouration seen in organisms as diverse as beetles, butterflies and frogs. Birds are notoriously colourful, with a palette stretching from the UV (ultraviolet) to deep red, and understanding why they have evolved such diverse colours, particularly in taxa with remarkably large colour diversity such as hummingbirds (Trochilidae) and sunbirds (Nectariniidae) is slowly growing (Gruson et al., 2021; Nicolaï et al., 2024). One proximate explanation is that bird plumage can be derived from a single, or a mixture of colour producing mechanisms (i.e. pigments and/or structural colours) distributed across the body. This can result in either a single colour across the body or multiple patches of different colours (Mason & Bowie, 2020). This complexity in colouration increases contrast among body regions and thus potentially signal efficacy in different environments. In addition, it also enables different (possibly competing) selective forces, such as natural selection and sexual selection, to shape colouration of different feather patches, thereby minimizing potential trade‐offs between the effects of these two forces on phenotype (Beltrán et al., 2021; Cooney et al., 2019; Doucet et al., 2007; Endler, 1990; Endler, 1992; Friedman & Remeš, 2024; Gruson et al., 2021; Simpson et al., 2020). Specifically, dorsal colours are more visible to predators and might thus be under natural selection for camouflage, while ventral colours are mostly hidden from the view of predators and therefore might be more subject to sexual selection (Gruson et al., 2021; Nicolaï et al., 2024; Simpson et al., 2020). Some colours, such as iridescence, can function for both crypsis and signalling, due to their high directionality of signal propagation (Simpson & McGraw, 2018). For example, iridescence in male sunbirds is mostly found on the dorsum where they are cryptic unless viewed from a certain angle. Bright carotenoid‐based colours (yellow, red) are mostly located on ventral surfaces, where they are concealed and exposed only during display (Nicolaï et al., 2024). As such, when investigating colour evolution, it is necessary to understand the complexity of different colour mechanisms, whether pigment‐ or structure‐based, in producing the overall phenotype.

Sunbirds and spiderhunters (Nectariniidae) are a large (125–145 species) family with some of the most spectacularly coloured plumages in class Aves (Bowie & Fjeldså, 2020). Plumage colouration differs substantially within, as well as among, sunbird genera. This diversity in colour is the result of several colour‐producing mechanisms, resulting in the variable expression of colour across several plumage regions and a complex phenotype in these nectarivorous birds (Nicolaï et al., 2024). For example, while spiderhunters (genus *Arachnothera*) are dull olive‐green and sexually monomorphic, in most other genera of sunbirds the sexes are dimorphic in colour with females having drab colours relative to brightly plumaged males. Males often combine bright iridescent colouration with intense carotenoid colouration, as well as darker melanin‐based colouration and unpigmented whites (Cheke et al., 2001). Since there is such diversity across species, sexes and body patches, sunbirds form an ideal model system to investigate how different selective forces influence colouration.

We have previously argued that the evolution of novel colour mechanisms, as well as sexual dichromatism, has promoted the diversification of sunbirds. That sexual dichromatism may promote lineage diversification suggests that the evolution of sunbird colouration might be influenced by sexual selection (Nicolaï et al., 2024). However, if sexual selection selected for honest signals, we would expect convergence towards the same signals, both in the colours produced (i.e. the signal) and the location where these signals occur (i.e. the visibility of this signal) (Prum, 1997). This is not true in sunbirds, where the evolution of colours is mostly divergent (Nicolaï et al., 2024). It thus remains unclear why species differ in colour.

Two factors have frequently been proposed to explain variation in interspecific (plumage) colour: the extent to which geographical ranges overlap and differences in light environment. The former can affect colours in two different ways. Isolation by distance (Wright, 1943) assumes that gene flow becomes increasingly restricted with increasing geographical distance. As such, a positive correlation between colour differences and geographical distance is expected. However, when gene flow is not restricted, prezygotic isolation could result in colour differences evolving as a communication signal to minimize the risk of hybridization, a process called reproductive character displacement (Anderson & Weir, 2021; Kirschel et al., 2020). In such a case, higher differences in colouration are expected among species with higher probabilities of hybridization. These can be closely related (i.e. recently diverged) species pairs that have not had sufficient time to accumulate reproductive incompatibilities (post‐zygotic), or species pairs with more interactions, that is, those whose distributional ranges overlap to a greater degree in sympatry. Both positive and negative effects of geographical distance on colour differences have been recovered in birds (Alatalo et al., 1994; Gruson et al., 2021; Kiere et al., 2009; Paulo et al., 2023; Saetre et al., 1993, 1997; Vallin et al., 2012). The influence of geographical distance is not exclusive to colouration, but applies to other signals as well, such as song (Anderson & Weir, 2021; Benedict & Bowie, 2009; Simpson et al., 2021).

As colours are not equally visible in all habitats, differences in colour might reflect differences in habitat use, with selection operating such that colours are either conspicuous or hidden. More specifically, isolation by light environment suggests that interspecific variation arises when species occupy different light environments with colours evolving to enhance signal efficiency (Endler, 1993; Schultz & Burns, 2013, 2017). Therefore, species with substantial overlap in habitat type should share a more similar colour palette than related lineages occupying different habitats, whereby selection for conspicuousness or crypsis (when this is the primary function of colouration) among different habitats would drive colour evolution. More precisely, to be more conspicuous in closed, dark habitats, species are expected to be brighter and show higher colour contrasts and more long‐wavelength colours (e.g. oranges and reds), than species living in open habitats (Endler, 1993; Gomez & Théry, 2004). These conspicuous colours are mostly expected in males, who are more likely to be under sexual selection. Conversely, to be as cryptic as possible, species tend to be darker in darker habitats (Endler, 1993; Marchetti, 1993), a pattern that we expect to be present mostly in dorsal patches, or in females since they are more exposed to predation during incubation (Cheke et al. (2001); but see Rogalla et al. (2022) showing that even males might engage in incubation). However, given the diversity of colours in sunbirds, it is possible that conspicuousness requires less convergence than crypsis (although this warrants further research). In at least a few clades (e.g. *Phylloscopus*, Pipridae, Coraciiformes) plumage colours fulfil these predictions (Babarović et al., 2023; Doucet et al., 2007; Endler & Théry, 1996; Gomez & Théry, 2004; Heindl & Winkler, 2003a, 2003b; Marchetti, 1993; Marcondes & Brumfield, 2019; McNaught & Owens, 2002; Schultz & Burns, 2017; Simpson & McGraw, 2018). In non‐avian taxa, there is also support for the association between variation in colour patterns and variation in the light environment in taxa as diverse as fish (Kranz et al., 2018), reptiles (Marshall et al., 2015; McLean et al., 2015) and beetles (Théry et al., 2008). However, divergence or convergence in colouration might differ among patches where colour serves different functions (i.e. crypsis vs. conspicuousness). While the light environment is of particular importance for visual signals, natural and sexual selection might result in similar patterns of conspicuousness and inconspicuousness in other signals, such as vocalizations adapting to the ambient soundscape (Boncoraglio & Saino, 2006).

A third hypothesis, the null model, postulates that differences in colouration result from genetic drift through time. In this case, we predict that differences in colour accumulate as a function of time since lineage divergence (i.e. genetic divergence). Similarly, range expansion takes time, genetic drift through time might also predict a correlation between colour divergence and distance across the landscape (in addition to a correlation with phylogenetic distance). Such correlations have been observed in birds, and also in other organisms such as frogs, fish and other taxa (Clark et al., 2022; Kirschel et al., 2020; Martin & Mendelson, 2012).

Here we use a phylogenetic comparative framework and ecological niche modelling to test hypotheses on the evolution of colour diversity in Nectariniidae. To do so, we use colour measurements of almost 85% of the extant species and test three hypotheses on how and why colour evolved across different patches. We ask if colouration is influenced by differences among species in: (1) geographical distance; (2) differences in light environment, or (3) whether colour has evolved over time in a pattern consistent with the expectations of genetic drift.

## 2 MATERIALS AND METHODS

### 2.1 Geographical distance and quantification of sympatry

We obtained distributional range maps for all sunbirds from BirdLife International (datum: WGS1984) (BirdLife International, NatureServe, 2012) and calculated the minimal distance between distribution ranges and the extent of overlap of distribution ranges. We used the ‘st_distance’ function of the sf package in R (Pebesma, 2018) to calculate the minimal distance between distribution ranges for all species pairs. In the case that the minimal distance between distribution ranges of a species pair was equal to zero, that is, sympatric in at least part of their range, hereafter referred to as a ‘sympatric pair’ for simplicity, we used the ‘st_intersection’ function of the sf package in R to calculate the degree of overlap between distribution ranges of every sympatric species pair. Unless the distribution ranges of two species are equal in size, overlap in distribution ranges is asymmetric: we calculated overlap in both directions by dividing the area overlapped by the distribution area of species 1 and species 2 to calculate the degree of sympatric overlap for each species.

### 2.2 Reflectance measurements

Reflectance measurements follow Nicolaï et al. (2024). In brief, reflectance spectra of 245 specimens of 106 species (60–85% of sunbird species, depending on the classification scheme adopted) were measured at the Royal Belgian Institute of Natural Sciences (RBINS), Royal Museum for Central Africa (RMCA) and The Field Museum (Data S1) (male 1 to 3 specimens per species, average 2.4; female 1 to 3 specimens per species, average 2.0). We used an AvaSpec‐ULS2048 L StarLine Versatile Fibre‐optic Spectrometer UV–VIS (300‐700 nm) (calibrated with a BS‐2 2% black and WS‐2 99% white standard) with an AvaLight‐DH‐S Deuterium‐Halogen Light Source to measure the reflectance of six body regions: crown, mantle, throat, upper and lower breast band (breast band 1 and 2 throughout the text), and belly (total number of averaged measurements = 1295). We connected the fibre optic cable with a reflection probe holder that was held at an angle of 90° placed directly on the patch, with the probe positioned at 0.5 cm from the bird. For iridescent patches we measured at the angle of maximal reflection. Given that colour of older specimens (>50 years) (Armenta et al., 2008) might have changed, we took precautionary measures (as outlined in Doucet & Hill, 2009) and excluded specimens that showed physical damage and dust. Birds vary in colour perception abilities, with some species being able to see UV (ultraviolet), and this might influence how colours (and differences) are perceived. To correct for this, we converted reflectance spectra into relative cone stimuli using an avian colourspace model (Maia et al., 2013) that accounts for how colours are perceived by birds (protocol following Nicolaï et al., 2024). An averaged avian UV visual system (Nectariniidae have UVS vision; Ödeen & Håstad, 2010) was used to simulate UV vision, and allowed us to calculate stimuli under idealized illumination using the R package ‘pavo’ (Maia et al., 2019). Cone stimulation values were used separately to calculate just‐noticeable differences (JNDs), or colour distances for each colour patch between all species pairs, both sympatric and allopatric, using standard settings in pavo (Maia et al., 2019). JNDs represent how colour is perceived by organisms, incorporating information on organismal visual systems. JND values below <1 are not perceived as different, whereas differences with JND > 1 are. Higher JND values thus correspond to higher colour divergence. Given that colours were converted to avian colour space, here they represent how differences are perceived by birds. Sympatric colour distances thus correspond to the colour distance between (and only between) all sympatric species pairs and are a metric for colour differences. To obtain a metric for whole‐body colourfulness, we calculated a whole‐body average JND‐colour distance matrix by averaging colour distances between species across all patches. All analyses were performed separately for males and females.

### 2.3 Ecological niche modelling and quantification of niche overlap

To construct a metric of light environment overlap, we first built ecological niche models (ENMs) of 106 sunbird species using Maxent ver. 3.4.1 (Phillips et al., 2017). The algorithm used by Maxent makes use of species presence records and environmental variables obtained from downscaled global climate models to predict a species range (Phillips et al., 2006). To collect sunbird occurrence records, we searched ebird (https://ebird.org/home), GBIF (GBIF.org, 2021), VertNet (http://vertnet.org/) and iDigBio (https://www.idigbio.org/), using the spocc package (Chamberlain et al., 2021) in the R 4.0.3 environment (R Core Team, 2018). We removed duplicate localities from the occurrence records of each species, and used DIVA‐GIS 7.5 (Hijmans et al., 2001) to map records, after which obvious outlier localities were removed from the dataset. In total we had an average of 1420 distribution records per species. We compared the occurrence records of each species with its range map from Birdlife International to assess the spatial representativeness of the distribution records within the species range. We found that the distribution records adequately cover the whole range of each species, suggesting that the occurrence data are not biased towards a specific region of the species range.

We used the average summer normalized difference vegetation index (NDVI) variable, when vegetation is at its peak, as a metric for light environment in the ENMs. Environmental layers were obtained at a 2.5‐min spatial resolution (this is about 4.5 km at the equator). Therefore, distribution records were thinned to 5 km. ENM performance was assessed using the area under the curve (AUC) metric of the receiving operator characteristic (ROC) curve (Swets, 1988). The ROC plot was created by selecting 80% of the data for training, and 20% of the data for testing: AUC values close to 0.5 suggests that the model has no predictive ability whereas values close to 1 show perfect predictive ability of the model (Guisan et al., 2017). AUC values for all models were above 0.8 suggesting good predictive performance of the models. We used Schoener's D niche overlap metric (Schoener, 1968), implemented in ENMtools 1.4.4. (Warren et al., 2010), to quantify the degree of niche overlap between each pair of species. Schoener's D ranges from 0 (no overlap; niches are completely different) to 1 (complete overlap; niches are identical) (Warren et al., 2010). We calculated Schoener's D for two different sets of variables. First, we calculated one value based on all environmental variables (Annual precipitation, annual temperature, topography (elevation and topographical heterogeneity)), NDVI and Solar Radiation Index. Additionally, as forest cover influences the light environment, which may influence signal communication, we also calculated Schoener's D to determine the degree of NDVI niche overlap between each species pair alone. Finally, for each species we calculated the average NDVI value as a metric for the average light environment of the habitats that the species occupies.

### 2.4 Statistical analyses

We used the R package ‘nlme’ to perform multiple Generalized Least Squares regressions (Pinheiro et al., 2023). All analyses were performed separately for males and females. As individual patches can have separate functions, we ran analyses on all plumage patches separately, as well as averaged across the body (Mason & Bowie, 2020). In addition, we ran analyses split into three data partitions containing: (1) all species pairs (*n* = 3834); (2) only sympatric species pairs (*n* = 1188) and, (3) only allopatric species pairs (*n* = 2636). We used these three datasets to test whether there is a correlation between colour divergence and three different predictor variables, fitted together (without interactions): (1) phylogenetic distance, that is the divergence time obtained from the recently estimated sunbird maximum clade credibility (MCC) tree (Bowie & Fjeldså, 2020; Nicolaï et al., 2024); (2) light environmental overlap, that is, NDVI overlap; and (3) degree of sympatry. We used the R package ‘car’ (Fox & Weisberg, 2019) to calculate VIF (variance inflation factors) to investigate multicollinearity among predictor values and found that all VIF scores were below 1.25, suggesting limited collinearity (Table S1). In sympatric species, overlap values are larger than 0 (i.e. when overlap is 0, they are not sympatric) but minimal distances between distribution ranges equal to 0 (i.e. when minimal distances are larger than 0, species are not sympatric). Similarly, in allopatric species, overlap values between ranges are equal to 0, but minimal distances are larger than 0. As a result, within the framework of our hypotheses, ‘overlap’ (but also ‘sympatry’), will have effects in opposite directions to ‘minimal distance’, even though the same hypothesis is tested (i.e. how is a metric of geographical distance related to colour divergence). Thus, in the analyses using all taxa (both sympatric and allopatric), sympatry was treated as a categorical (yes/no) variable. In the sympatric only dataset, the degree of species range overlap was used. In the allopatric only dataset the minimal distance between species ranges was used.

Finally, if light environment is associated with colour divergence, then colour divergence may be a way for individuals to optimize conspicuousness in different habitats. We used a phylogenetic generalized least squares (PGLS) model (implemented using caper (Orme et al., 2012), with and without lambda being optimized using ML), to analyse variation in plumage colour variables in relation to average species NDVI values. To do so we used the package pavo (Maia et al., 2019) to calculate brightness (B2, mean brightness—the sum of relative reflectance over the entire spectral range), hue (H1, peak wavelength hue—the wavelength of maximum reflectance) and chroma (S1, the relative contribution of a spectral range to the total brightness) across multiple parts of the light spectrum: S1U (300–400 nm, Ultraviolet), S1V (300–415 nm, Violet), S1B (400–510 nm, Blue), S1G (510–605 nm, Green), S1Y (550–625 nm, Yellow), S1R (605–700 nm, Red). Analyses were run separately for males (*n* = 82) and females (*n* = 75).

To correct for potential false discovery rate (FDR) due to the large number of tests being performed, we used *p.adjust* in the ‘stats’ package using the method suggested by Benjamini and Hochberg (1995) on all analyses.

## 3 RESULTS

**FIGURE 1:**
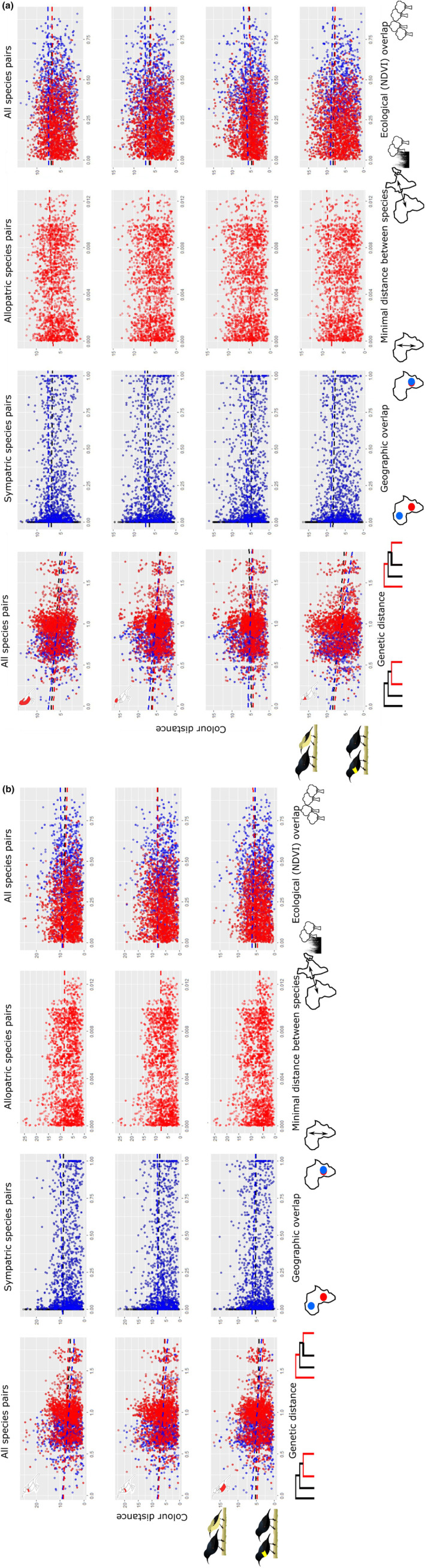
(a) Male colour distance as a function of phylogenetic distance, geographical overlap, minimal distance between species and ecological overlap for all body patches averaged, the crown, the mantle and the throat. Linear regressions shown use coefficients of GLS. (b) Male colour distance as a function of phylogenetic distance, geographical overlap, minimal distance between species and ecological overlap for breastband 1, breastband 2 and belly. Linear regressions shown use coefficients of GLS.

**FIGURE 2:**
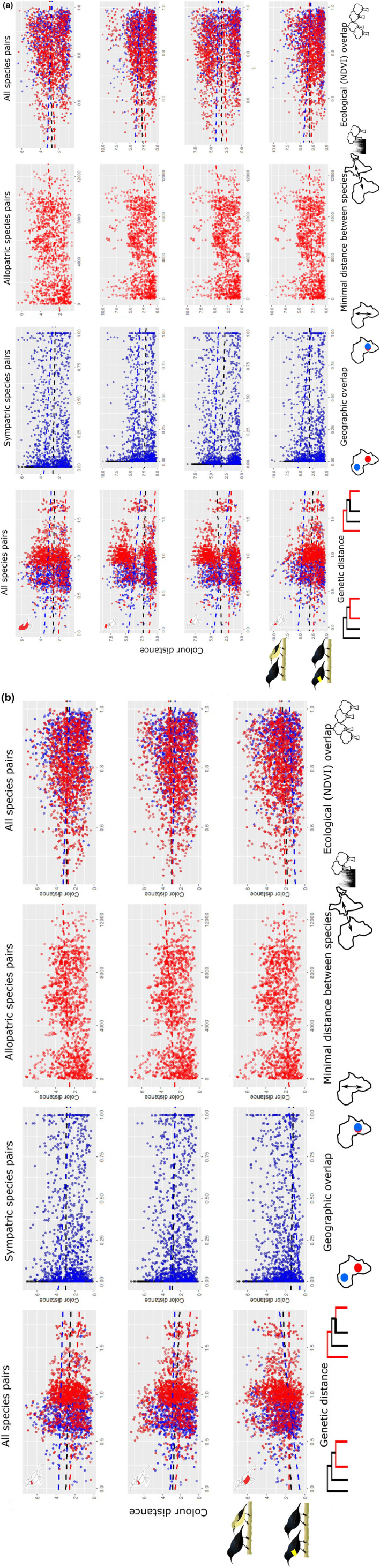
(a) Female colour distance as a function of phylogenetic distance, geographical overlap, minimal distance between species and ecological overlap for all body patches averaged, the crown, the mantle and the throat. Linear regressions shown use coefficients of GLS. (b) Female colour distance as function of phylogenetic distance, geographical overlap, minimal distance between species and ecological overlap for breastband 1, breastband 2 and belly. Linear regressions shown use coefficients of GLS.

Differences in colouration among sunbird taxa are explained by different drivers at multiple hierarchical levels, including differences between sympatric and allopatric species pairs, between males versus females and between different patches on an individual (Tables 1 and 2, Figures 1 and 2). Even though many predictor values show a significant pattern, the amount of variance explained appears to be low for all models tested presumably because untested variables co‐explain patterns observed.

**TABLE 1:** Results of the generalized least squares (gls) analyses between colour divergence in **males**, phylogenetic distance, NDVI overlap and different metrics of sympatry.

		All taxa		Sympatric taxa		Allopatric taxa	
		Effect size	*p*‐value	Effect size	*p*‐value	Effect size	*p*‐value
Average	Phylogenetic distance	−1.01	**<.01**	−1.92	**<.01**	−1.02	**<.01**
	Sympatry/geographical overlap/min. distance	−0.17	.11	−0.79	**<.01**	0.06	**<.01**
	NDVI overlap	−0.17	.53	0.16	.7	0.2	.59
Crown	Phylogenetic distance	−1.2	**<.01**	−1.76	**<.01**	−1.19	**<.01**
	Sympatry/geographical overlap/min. distance	0.4	**<.01**	−0.67	**.03**	0.04	**.05**
	NDVI overlap	−0.2	.54	0.21	.7	−0.04	.92
Mantle	Phylogenetic distance	0.33	.14	−0.6	.24	0.04	.92
	Sympatry/geographical overlap/min. distance	0.13	.37	−1.41	**<.01**	0.12	**<.01**
	NDVI overlap	0.61	.07	1.29	**.02**	1.29	**<.010**
Throat	Phylogenetic distance	−1.63	**<.01**	−3.13	**<.01**	−1.66	**<.01**
	Sympatry/geographical overlap/min. distance	−0.57	**<.01**	−0.13	.7	0.09	**<.01**
	NDVI overlap	−0.56	.14	−0.67	.25	−0.06	.92
Breastband 1	Phylogenetic distance	−1.66	**<.01**	−2.64	**<.01**	−1.13	**<.01**
	Sympatry/geographical overlap/min. distance	−0.21	.29	−1.8	**<.01**	−0.03	.31
	NDVI overlap	−0.98	**.04**	0.92	.25	−1.67	**<.01**
Breastband 2	Phylogenetic distance	−1.39	**<.01**	−1.94	**<.01**	−1.3	**<.01**
	Sympatry/geographical overlap/min. distance	−0.65	**<.01**	−0.92	**.02**	0.02	.47
	NDVI overlap	0.02	.96	0.12	.84	0.44	.47
Belly	Phylogenetic distance	−0.51	**.01**	−1.43	**<.01**	−0.89	**<.01**
	Sympatry/geographical overlap/min. distance	−0.11	.37	0.19	.63	0.12	**<.01**
	NDVI overlap	0.09	.76	−0.91	.11	1.25	**<.01**

*Note*: In sympatric species, overlap values are larger than 0 but minimal distances are equal to 0. Similarly, in allopatric species overlap values are equal to 0 but minimal distances are larger than 0. As such different metrics were used to quantify sympatry. In the analyses using all taxa (both sympatric and allopatric), the categorical variable sympatric species (yes/no) was used. In the sympatric taxa only dataset, the degree of range overlap was used. In the allopatric taxa only dataset, the minimal distance between species was used. ‘Sympatry’ and ‘overlap’ are expected to result in effects with the same signal (i.e. sympatric species haver higher overlap), while ‘minimal distance’ should have an effect in the opposite direction (e.g. sympatric species have lower minimal distances).

**TABLE 2:** Results of the generalized least squares (gls) analyses between colour divergence in **females**, phylogenetic distance, NDVI overlap and different metrics of sympatry.

		All taxa		Sympatric taxa		Allopatric taxa	
		Effect size	*p*‐value	Effect size	*p*‐value	Effect size	*p*‐value
Average	Phylogenetic distance	−0.07	.57	−0.4	.11	−0.44	**<.01**
	Sympatry/geographical overlap/min. distance	−0.23	**<.01**	0.19	.22	0	**<.01**
	NDVI overlap	0.3	.21	−0.83	.13	0.93	**<.01**
Crown	Phylogenetic distance	−0.2	.21	−1	**<.01**	−0.49	**<.01**
	Sympatry/geographical overlap/min. distance	−0.24	**<.01**	0.29	.21	0	**<.01**
	NDVI overlap	0.95	**<.01**	−1.35	.1	1.99	**<.01**
Mantle	Phylogenetic distance	0.38	**.03**	−1.32	**<.01**	−0.2	.3
	Sympatry/geographical overlap/min. distance	−0.6	**<.01**	0.18	.42	0	**<.01**
	NDVI overlap	−0.07	.87	−0.67	.41	0.88	**.03**
Throat	Phylogenetic distance	−0.34	**.02**	−0.67	.09	−0.51	**<.01**
	Sympatry/geographical overlap/min. distance	−0.01	.87	0.34	.17	0	**<.01**
	NDVI overlap	−0.08	.87	−2.34	**<.01**	0.74	**.03**
Breastband 1	Phylogenetic distance	−0.27	**.01**	−0.22	.4	−0.56	**<.01**
	Sympatry/geographical overlap/min. distance	−0.05	.53	0.11	.48	0	**<.01**
	NDVI overlap	−0.13	.65	−1.15	.08	0.34	.17
Breastband 2	Phylogenetic distance	−0.42	**<.01**	−0.27	.31	−0.87	**<.01**
	Sympatry/geographical overlap/min. distance	−0.26	**<.01**	0.06	.67	0	**<.01**
	NDVI overlap	0.21	.52	−0.51	.39	0.68	**.01**
Belly	Phylogenetic distance	0.45	**<.01**	1.1	**<.01**	0	.98
	Sympatry/geographical overlap/min. distance	−0.21	**<.01**	0.17	.25	0	**<.01**
	NDVI overlap	0.91	**<.01**	1.02	.08	0.94	**<.01**

*Note*: In sympatric species, overlap values are larger than 0 but minimal distances are equal to 0. Similarly, in allopatric species overlap values are equal to 0 but minimal distances are larger than 0. As such different metrics were used to quantify sympatry. In the analyses using all taxa (both sympatric and allopatric), the categorical variable sympatric species (yes/no) was used. In the sympatric taxa only dataset, the degree of range overlap was used. In the allopatric taxa only dataset, the minimal distance between species was used. ‘Sympatry’ and ‘overlap’ are expected to result in effects with the same signal (i.e. sympatric species have higher overlap), while ‘minimal distance’ should have an effect in the opposite direction (e.g. sympatric species have lower minimal distances). Significant (*p* < .05) values are shown in bold.

### 3.1 Colour distances and phylogenetic distances

In males (Table 1, Figure 1), except for the mantle, increases in phylogenetic distances are correlated with decreases in colour distance, that is, closely related species are more likely to look different.

In females (Table 2, Figure 2), results for different patches are more variable. When comparing all species pairs, increases in phylogenetic distances are correlated to decreased colour differences in the throat, breastband 1 and 2, while correlated to increased colour differences for the mantle and belly. When comparing sympatric species pairs only, increases in phylogenetic distances are correlated to decreased colour distances in the crown, mantle and belly. When comparing allopatric species pairs, an increase in genetic differences is correlated to smaller colour differences in all patches except the belly.

### 3.2 Colour distances and geographical distances

In males, the effect of geographical overlap is patch specific (Table 1, Figure 1). In dorsal patches, increases in geographical overlap or decreases in minimal distance between species pairs are correlated to smaller colour distance, that is, co‐occurring species are more likely to look alike (Table 1, Figure 1). In the throat, this effect is only significant when considering all species pairs and allopatric species pairs. In breastband 1, this effect is present only in sympatric species pairs, while in breastband 2 this pattern is present in all analyses except those considering allopatric species pairs. In the belly, this pattern is significant only in allopatric species pairs (Table 1, Figure 1).

In females, when comparing across all species, an increase in geographical overlap is correlated with smaller colour differences in the crown, mantle, breastband 2 and belly (Table 2, Figure 2). When comparing sympatric species pairs only, increases in geographical overlap are correlated with decreased colour distances in the crown, mantle and belly, while for comparison among allopatric species pairs, an increase in minimal distances correlates to larger colour differences for all patches (Table 2, Figure 2).

### 3.3 Colour distances and ecological overlap

The effect of ecological (i.e. Schoener's D) overlap is patch specific, influencing colouration for only a few patches (Tables 1 and 2). In males, increases in ecological overlap correspond to increases in colour differences of the mantle (both sympatric and allopatric taxa) and belly (allopatric taxa), that is, species occurring in the same habitat have more different colours (Table 1, Figure 1). In breastband 1, the opposite effect is present, where allopatric species pairs, as well as all species pairs, show a significant decrease in colour distances with increasing ecological overlap.

In females, when comparing across all species, increases in ecological overlap are associated with larger colour distances for the belly and crown (Table 2, Figure 2). When comparing allopatric species pairs, there was no correlation between colouration and ecological overlap. When comparing sympatric species pairs only, increases in ecological overlap are correlated with decreased colour differences on the throat. Finally, an increase in ecological overlap corresponds to increased colour differences for all patches except breastband 1.

### 3.4 Colour metrics do not differ among light environments

Brightness was never correlated with NDVI values (Tables 3 and 4; Tables S2 and S3), with the exception of breastband 1, where higher brightness is correlated with closed habitats, but only for analyses where lambda is estimated using ML (*p* < .05), and was not significant in the other analyses (*p* < .1). For female belly, hues are higher (more reddish) in sunbirds living in closed (i.e. higher NDVI values) versus open habitats (i.e. lower NDVI values), but this result is only recovered when using default lambda values (Tables 3 and 4; Tables S2 and S3). Similarly, there are no parts of the light spectrum that are significantly brighter in sunbirds in closed (i.e. higher NDVI values) versus open (i.e. lower NDVI values) habitats after correcting for multiple testing (Tables 3 and 4; Tables S2 and S3). Possible exceptions are blue and yellow chroma which are (almost) significant (*p* < .05 and <.07) for the mantle of males, but not when lambda is estimated using ML (*p* < .14 and *p* < .44 respectively).

**TABLE 3:** Results of a PGLS relating NDVI to chroma (S1U‐R) S1U (300–400 nm), S1V (300–415 nm), S1B (400–510 nm), S1G (510–605 nm), S1Y (550–625 nm), S1R (605–700 nm) and hue (H1) for male colour.

	Estimate	Std. error	*t*‐val.	*p*‐val.	*p*‐val. Cor.
Crown					
S1U_1	−0.20502	0.410942	−0.49889	.619225	.785422
S1V_1	−0.23391	0.465425	−0.50258	.616643	.785422
S1B_1	0.003279	0.290269	0.011297	.991014	.991014
S1G_1	−0.36096	0.286529	−1.25976	.211418	.563782
S1Y_1	−0.36105	0.205818	−1.75424	.083219	.37119
S1R_1	0.556487	0.679579	0.818869	.415294	.785422
H1	−242.924	601.2026	−0.40406	.687244	.785422
B2	−32.1876	18.92102	−1.70116	.092798	.37119
Mantle					
S1U_1	−0.06367	0.296053	−0.21505	.830275	.984182
S1V_1	−0.03049	0.32522	−0.09376	.925534	.984182
S1B_1	−0.0034	0.170868	−0.01989	.984182	.984182
S1G_1	−0.42967	0.316054	−1.35947	.177818	.71127
S1Y_1	−0.36023	0.196498	−1.83323	.070488	.563908
S1R_1	0.493343	0.563292	0.875822	.383749	.984182
H1	10.39962	323.4948	0.032148	.974434	.984182
B2	2.543788	18.31504	0.138891	.889886	.984182
Throat					
S1U_1	−0.79425	0.358183	−2.21745	.02943	.117719
S1V_1	−0.96541	0.413689	−2.33365	.022125	.117719
S1B_1	−0.43688	0.287014	−1.52215	.131915	.150761
S1G_1	0.660207	0.359793	1.834963	.070228	.150761
S1Y_1	0.394411	0.256531	1.537478	.128122	.150761
S1R_1	0.569451	0.599066	0.950564	.344689	.344689
H1	872.0372	485.5759	1.795883	.076289	.150761
B2	−56.9959	35.40111	−1.61	.111336	.150761
Breast band 1					
S1U_1	0.20102	0.341278	0.589022	.557506	.920269
S1V_1	0.218031	0.387428	0.562766	.575168	.920269
S1B_1	−0.00336	0.405575	−0.00828	.993417	.993417
S1G_1	−0.04375	0.262114	−0.16691	.867864	.991844
S1Y_1	−0.18204	0.231233	−0.78727	.433451	.920269
S1R_1	−0.15693	0.609993	−0.25727	.797635	.991844
H1	−842.546	372.6755	−2.2608	.026488	.211903
B2	−30.551	31.09186	−0.9826	.328764	.920269
Breast band 2					
S1U_1	−0.41209	0.352677	−1.16847	.246088	.410768
S1V_1	−0.49905	0.39753	−1.25538	.212995	.410768
S1B_1	−0.44279	0.302448	−1.46401	.147111	.410768
S1G_1	0.021165	0.220733	0.095886	.923851	.923851
S1Y_1	0.138937	0.192156	0.723041	.471763	.539158
S1R_1	0.828585	0.613605	1.350357	.180709	.410768
H1	−531.226	465.0434	−1.14232	.25673	.410768
B2	−27.5928	35.83644	−0.76997	.443588	.539158
Belly					
S1U_1	−0.57239	0.323127	−1.77142	.080299	.128479
S1V_1	−0.67222	0.35957	−1.86952	.06521	.128479
S1B_1	−0.62227	0.254694	−2.44321	.016759	.067036
S1G_1	−0.04378	0.218723	−0.20017	.841855	.841855
S1Y_1	0.132805	0.166618	0.797059	.427776	.570368
S1R_1	1.232586	0.571412	2.15709	.034	.090667
H1	1072.302	315.1368	3.402656	.001045	**.008358**
B2	−22.8643	42.62927	−0.53635	.593203	.677947

*Note*: Significant (*p* < .05) values are shown in bold.

**TABLE 4:** Results of a PGLS relating NDVI to chroma (S1U‐R) S1U (300–400 nm), S1V (300–415 nm), S1B (400–510 nm), S1G (510–605 nm), S1Y (550–625 nm), S1R (605–700 nm) and hue (H1) for female colour.

	Estimate	Std. error	*t*‐val.	*p*‐val.	*p*‐val. Cor.
Crown					
S1U_1	0.017078	0.286658	0.059578	.952662	.952662
S1V_1	0.030081	0.304997	0.098628	.921716	.952662
S1B_1	0.21609	0.147209	1.467912	.146607	.580112
S1G_1	−0.01398	0.111593	−0.12531	.900639	.952662
S1Y_1	−0.11811	0.079783	−1.48033	.143272	.580112
S1R_1	−0.21954	0.34708	−0.63254	.529094	.846551
H1	−134.977	200.2498	−0.67405	.502503	.846551
B2	58.46488	46.98641	1.244293	.217542	.580112
Mantle					
S1U_1	−0.57832	0.345508	−1.67383	.098626	.157802
S1V_1	−0.66592	0.380302	−1.75102	.084323	.157802
S1B_1	−0.52913	0.186094	−2.84333	**.005847**	**.046778**
S1G_1	0.065875	0.087167	0.75573	.452347	.574442
S1Y_1	0.190763	0.077198	2.4711	**.015907**	.063627
S1R_1	1.036912	0.542038	1.912986	.059841	.157802
H1	−102.349	238.5703	−0.42901	.669233	.669233
B2	17.06417	25.32405	0.673832	.502637	.574442
Throat					
S1U_1	−0.20247	0.295761	−0.68458	.495871	.661162
S1V_1	−0.2326	0.3276	−0.71001	.480056	.661162
S1B_1	−0.06008	0.185393	−0.32408	.746845	.746845
S1G_1	−0.14831	0.112894	−1.31374	.193222	.621506
S1Y_1	−0.04413	0.088228	−0.50024	.618478	.706832
S1R_1	0.407088	0.466732	0.87221	.386076	.661162
H1	−647.511	355.5183	−1.82132	.072832	.582656
B2	45.90405	38.1609	1.202908	.233065	.621506
Breast band 1					
S1U_1	−0.08177	0.295956	−0.27629	.783138	.884916
S1V_1	−0.08335	0.314101	−0.26535	.79152	.884916
S1B_1	0.023274	0.16021	0.145268	.884916	.884916
S1G_1	−0.0275	0.137348	−0.20019	.841914	.884916
S1Y_1	−0.03928	0.110965	−0.35398	.724421	.884916
S1R_1	0.084917	0.356396	0.238267	.81237	.884916
H1	61.6888	86.671	0.711758	.47898	.884916
B2	93.44479	36.42598	2.565334	.012452	.09962
Breast band 2					
S1U_1	0.013543	0.303011	0.044693	.964479	.964479
S1V_1	0.039014	0.318447	0.122513	.902844	.964479
S1B_1	0.152819	0.175853	0.869019	.387807	.678448
S1G_1	−0.10329	0.12845	−0.80415	.42403	.678448
S1Y_1	−0.11682	0.112976	−1.03403	.304679	.678448
S1R_1	−0.06402	0.303475	−0.21097	.833527	.964479
H1	135.8813	87.74579	1.548579	.125992	.50397
B2	86.42515	48.94804	1.765651	.081815	.50397
Belly					
S1U_1	−0.33244	0.285412	−1.16476	.248128	.57004
S1V_1	−0.32043	0.289846	−1.10553	.272768	.57004
S1B_1	0.123049	0.192649	0.638721	.525119	.600136
S1G_1	0.108808	0.117615	0.925121	.358127	.57004
S1Y_1	0.097167	0.097698	0.994559	.323426	.57004
S1R_1	0.103193	0.214407	0.481296	.631829	.631829
H1	87.32017	109.4051	0.798136	.42753	.57004
B2	113.5255	73.02572	1.554595	.124618	.57004

*Note*: Significant (*p* < .05) values are shown in bold.

## 4 DISCUSSION

Colouration in birds comprises a complex of different colour patches that are individually and collectively under varying degrees and forms of natural and sexual selection (Gruson et al., 2021; Marcondes & Brumfield, 2019; Schultz & Burns, 2017). This is reflected in our results, where different patches respond differently to geographical, ecological and genetic divergence.

In both females and males, colour differences are not likely the result of genetic drift of colour genes. Indeed, in most cases when a relationship between phylogenetic distance and colouration was found, mostly in the allopatric only dataset, it was negative, that is, more closely related species show more divergent colours.

In females, the relationship between genetic and colour divergence was less congruent between patches and datasets. We found no relationship between phylogenetic distance and colouration in most of our sympatric species pair analyses. These results are similar to Paulo et al. (2013), who found no relationship between colour and genetic diversity in manakins. In a few cases, phylogenetic distance and colour distance were positively correlated, more specifically in the mantle and belly when using sympatric and all species pairs.

In males, but not females, more closely related species had more divergent colours, a significant trend in almost all patches. These suggest that co‐occurring males are selected to be as different as possible, aiding in species recognition. As such, these results are consistent with sexual selection, confirming previous findings showing that sexual dichromatism and the evolution of novel colours promote diversification of sunbirds (Nicolaï et al., 2024). In this context, our results are consistent with the formation of pre‐zygotic barriers, the initial stage of reproductive isolation (i.e. hybridization avoidance), in facilitating lineage diversification in sunbirds.

However, a key prediction of the hybridization avoidance hypothesis is that species with greater geographical overlap should be more different, is not supported in our dataset. In both the male and female datasets, we find that higher minimal distances are correlated with more different colours. This finding is consistent with the isolation by distance hypothesis (Wright, 1943) since we found that colours are more similar in sympatric species with higher overlap and allopatric species with smaller distances between them. Similar findings have been previously recorded in other bird clades (McNaught & Owens, 2002: Laaksonen et al., 2015; Miller et al., 2019; Simpson et al., 2021).

The mechanisms underlying this pattern, where colours are more similar in species that occur closer to each other, are unclear. One reason might be that the number of colours attainable is limited when many species co‐occur, forcing sympatric species to be similar. However, given the multiple colour mechanisms present in sunbirds, this seem an unlikely mechanism (Nicolaï et al., 2024).

Alternatively, species identification might be so efficient or hybridization in general so unlikely, that colouration is only important between closely related species. It is possible that previously allopatric taxa can come into secondary contact without hybridizing only when mating traits such as colouration are sufficiently differentiated to prevent interbreeding (Templeton, 1981). In such cases, geographical overlap might not be a good proxy for hybridization and while phylogenetic distance would still be a predictor of colour divergence, sympatric overlap would not. Consistent with the above is the rarity of sunbird hybridization (Cheke et al., 2001; McEntee et al., 2016). This, together with the strong negative effect of genetic distance on colour differences, makes gene flow as a potential explanation for interspecific colour differences very unlikely.

Current distributions of species may be a poor approximation of species ranges near the time of lineage divergence when hybridization was more likely. In this case, patterns between colouration and the degree of geographical overlap might be difficult to recover (Losos & Glor, 2003). Indeed, many Indo‐Pacific sunbird species have distributions across multiple islands, suggesting that their current distribution is a product of dispersal after lineage divergence (but see Warren et al., 2003).

Finally, while geographical distances were calculated based on the entire species range, colours were measured using a few specimens from different localities. While some information on subspecies differences in colours exists, intraspecific variation in colouration has not been described in detail, making it difficult to assess how this would influence the results.

In addition to genetic and geographical divergence, we predicted that environmental divergence might correlate with colour divergence. More specifically, the light environment hypothesis predicts that animals occupying similar light environments, should have similar colours to optimize signal transmission. In only one patch (breastband 1) of male allopatric species pairs did we recover such a signal of selection for convergent colouration in similar habitats. While sexual display is not well studied in sunbirds, some data suggest that male sunbirds use displays that involve leaning forward, projecting dorsal parts including the head, mantle and tail, sometimes accompanied with wing fluttering and presentation of pectoral tufts (Bowie & Fjeldså, 2020; Jensen et al., 2016; Raleigh, 2017; Skead, 1967; Tsang et al., 2008; Wellman & Downs, 2010). As such, breastband 1 is likely involved in sexual communication, making this result consistent with those in other bird taxa where patches used in sexual communication had more similar colours in similar habitats (Laaksonen et al., 2015; McNaught & Owens, 2002; Miller et al., 2019; Rohwer, 1973; Simpson et al., 2021; Weckstein, 2005). Similarly, in only one test, the throat in female sympatric species pairs was a negative correlation recovered between throat divergence and ecological overlap, suggesting that for this patch, when sympatry is established, species in the same habitat have more similar colours.

However, for all other patches where a relationship between colour differences and environmental overlap was found (mantle and belly in males; all patches in female allopatric species pairs and some patches in other datasets), the pattern was the opposite, that is, an increase in niche overlap resulted in increases in colour differences. This suggests that adaptation to the light environment might not be true at a large ecological scale (i.e. similar macrohabitats), but that at a smaller scale, for example, across a vertical gradient, light environment might influence sunbird colouration. However, we lack the data to test this hypothesis, but interestingly, both mantle and belly are expected to be most visible across a vertical gradient, that is, differing across the dorsoventral axis rather than the anteroposterior axis.

To further explore the potential influence of light environment on colouration in sunbirds we investigated how brightness and different parts of the light spectrum (i.e. S1 values in Tables 3 and 4) correlated with different degrees of forest cover (NDVI). We found no correlation between brightness and forest cover (similar to McNaught & Owens, 2002, but different from Marchetti, 1993 and Babarović et al., 2023), possibly because in sunbirds many patches are iridescent and thus both bright and dark, depending on the viewing angle. In such a case, species might have bright and conspicuous signals during communication that are dark in other scenarios. One exception might be breastband 1, where higher brightness is correlated with closed habitats which would increase consciousness (as seen in Babarović et al., 2023 and Marchetti, 1993). It is important to note that we only measured iridescent colours at their brightest.

No patches showed significant patterns in hue and chroma across all analyses. In female bellies, species occupying more forested habitats have hues (the peak wavelength) that are significantly higher (i.e. more reddish) but only when lambda was estimated using ML (*p* < .05 with fixed lambda, and *p* < .1 when lambda was optimized). This is in line with predictions of the light environment hypothesis (Endler, 1993; McNaught & Owens, 2002) and results in other bird clades where ventral patches were under greater sexual selection (Friedman & Remeš, 2024; Schultz & Burns, 2017; Marcondes & Brumfield, 2019). These results, even if limited to two patches, are consistent with previous work that conspicuous colours are expected ventrally, whereas more camouflaged (including iridescent) colours are expected dorsally. Finally, no parts of the light spectrum are significantly brighter in sunbirds from closed (i.e. higher NDVI values) versus open (i.e. lower NDVI values) habitats.

Interestingly, the belly was not previously thought to be involved in sunbird display. That environment influences colouration in at least one patch, and provides us with exciting future research avenues. Not much is known about sunbird display, so more information on how, and in which micro‐ and macro‐habitat sunbirds generally live and display, might further strengthen our results. A better quantification of the ‘openness’ of the display habitat would provide valuable information, as light conditions differ along a vertical gradient, making for example, the upper canopy of even dense forest quite bright (McNaught & Owens, 2002; Nilsson et al., 2022).

Better measurements of ‘openness’ would have important implications for most other studies, including research on non‐avian taxa, that currently use gross estimations of light environment and would further highlight the need for measuring species‐specific light environments. Furthermore, other environmental factors, outside the scope of this work, including UV radiation, temperature, latitude and elevation, are known to influence bird colouration (Martin et al., 2010, 2015; Nicolaï et al., 2020; Porter et al., 2023; Rogalla et al., 2022).

More generally, these results highlight the importance to explore variation in colouration, not as a single value, but as a complex of interacting colour patches—each of which is under different selective forces. While research has focussed on conspicuous colouration in birds, having bright colours (or other signals), as well as complex colour patterns is not limited to birds. However, by using sunbirds as a model system, we provide a framework for future research to test similar hypotheses in other taxa and signals that have modular structure. In particular, differences between dorsal and ventral colours are likely to differ in function, for example in another colourful group such as butterflies.

## AUTHOR CONTRIBUTIONS

**M. P. J. Nicolaï:** Conceptualization (lead); data curation (equal); formal analysis (lead); investigation (lead); methodology (lead); resources (lead); software (lead); visualization (lead); writing – original draft (lead); writing – review and editing (lead). **S. Rogalla:** Data curation (lead); investigation (equal); resources (equal); writing – original draft (equal); writing – review and editing (equal). **M. Yousefi:** Conceptualization (equal); formal analysis (equal); writing – original draft (equal); writing – review and editing (equal). **R. C. K. Bowie:** Data curation (lead); formal analysis (equal); supervision (equal); writing – original draft (equal). **L. D'Alba:** Data curation (equal); methodology (equal); resources (equal); supervision (equal); writing – review and editing (equal). **M. D. Shawkey:** Conceptualization (equal); data curation (equal); funding acquisition (lead); methodology (equal); supervision (equal); writing – original draft (lead); writing – review and editing (equal).

## FUNDING INFORMATION

This work was supported by Fonds Wetenschappelijk Onderzoek – Vlaanderen (G007117N, GOG2217N, G0E8322N), US Air Force Office of Scientific Research (FA9550‐18‐1‐0477), Bijzonder Onderzoeks Fonds UGent (01P06322, BOF.PDO.2022.0015.01) and Human Frontier Science Program (RGP0047).

## CONFLICT OF INTEREST STATEMENT

None declared.

## Supporting information


Data S1.

Table S1.

## Data Availability

Data and code are publically available at a repository: https://datadryad.org/stash/share/pZgc9H8P841zMPmhz‐94M9Jka2DSzWqvw4lDIlUvuaM
