# Supplementary material for: Ecological, genetic and geographical divergence explain differences in colouration among sunbird species (Nectariniidae)
Source: Ecol Evol. 2024 Sep 11;14(9):e11427. doi: 10.1002/ece3.11427 (PMC11387724; doi:10.1002/ece3.11427)
Supplement: Supplementary file 2 — Table S1. [file ECE3-14-e11427-s001.docx]

**Supplementary tables.**

Supplementary table 1. VIF scores for each of the predictor values used.

|  | Phylogenetic distance | Sympatry | NDVI overlap | Geographic overlap | Minimal distance |
| --- | --- | --- | --- | --- | --- |
| VIF score | 1.180 | 1.238 | 1.077 | 1.090 | 1.026 |

Supplementary table 2. Results of a PGLS (now with lambda optimized using ML) relating NDVI to chroma (S1U-R) S1U (300-400 nm), S1V (300-415 nm), S1B (400-510 nm), S1G (510-605 nm), S1Y (550-625 nm), S1R (605-700 nm) and hue (H1) for male colour.

|  | Estimate | Std. Error | t-val. | p-val. | p-val. cor. |
| --- | --- | --- | --- | --- | --- |
| **Crown** |  |  |  |  |  |
| S1U_1 | 0.025795 | 0.343842 | 0.075019 | 0.940386 | 0.940386 |
| S1V_1 | 0.056006 | 0.383622 | 0.145994 | 0.884293 | 0.940386 |
| S1B_1 | 0.117705 | 0.223458 | 0.526743 | 0.59983 | 0.799773 |
| S1G_1 | -0.47332 | 0.291766 | -1.62227 | 0.10868 | 0.50836 |
| S1Y_1 | -0.32478 | 0.21066 | -1.54171 | 0.12709 | 0.50836 |
| S1R_1 | 0.395547 | 0.560013 | 0.706317 | 0.482043 | 0.799773 |
| H1 | -335.224 | 495.4879 | -0.67655 | 0.50064 | 0.799773 |
| B2 | -15.0828 | 20.35702 | -0.74091 | 0.460915 | 0.799773 |
| **Mantle** |  |  |  |  |  |
| S1U_1 | 0.049869 | 0.288869 | 0.172637 | 0.863373 | 0.863373 |
| S1V_1 | 0.09042 | 0.31941 | 0.283085 | 0.777843 | 0.863373 |
| S1B_1 | -0.06729 | 0.175495 | -0.38346 | 0.702398 | 0.863373 |
| S1G_1 | -0.56471 | 0.32559 | -1.7344 | 0.086698 | 0.693584 |
| S1Y_1 | -0.24527 | 0.210007 | -1.1679 | 0.246314 | 0.817967 |
| S1R_1 | 0.442282 | 0.547031 | 0.808513 | 0.421193 | 0.842387 |
| H1 | -80.6105 | 350.0181 | -0.2303 | 0.818444 | 0.863373 |
| B2 | 18.25303 | 17.7444 | 1.028664 | 0.306738 | 0.817967 |
| **Throat** |  |  |  |  |  |
| S1U_1 | -0.43599 | 0.326836 | -1.33396 | 0.186001 | 0.464226 |
| S1V_1 | -0.48709 | 0.374655 | -1.30011 | 0.197298 | 0.464226 |
| S1B_1 | -0.08431 | 0.285392 | -0.29542 | 0.768441 | 0.878218 |
| S1G_1 | 0.033694 | 0.335605 | 0.100398 | 0.92028 | 0.92028 |
| S1Y_1 | 0.143488 | 0.240885 | 0.595669 | 0.553078 | 0.878218 |
| S1R_1 | 0.264189 | 0.619147 | 0.426698 | 0.670745 | 0.878218 |
| H1 | 513.9125 | 426.8129 | 1.20407 | 0.232113 | 0.464226 |
| B2 | 39.45648 | 32.40955 | 1.217434 | 0.227018 | 0.464226 |
| **Breast band 1** | |  |  |  |  |
| S1U_1 | 0.238567 | 0.307973 | 0.774636 | 0.440838 | 0.656188 |
| S1V_1 | 0.246679 | 0.357464 | 0.69008 | 0.492141 | 0.656188 |
| S1B_1 | -0.36725 | 0.359945 | -1.0203 | 0.310662 | 0.656188 |
| S1G_1 | 0.329542 | 0.219627 | 1.500464 | 0.137431 | 0.549723 |
| S1Y_1 | 0.358884 | 0.20373 | 1.761564 | 0.081964 | 0.549723 |
| S1R_1 | 0.436758 | 0.568496 | 0.76827 | 0.444589 | 0.656188 |
| H1 | -142.815 | 390.608 | -0.36562 | 0.715612 | 0.715612 |
| B2 | -12.4545 | 31.89371 | -0.3905 | 0.697204 | 0.715612 |
| **Breast band 2** | |  |  |  |  |
| S1U_1 | -0.14914 | 0.293553 | -0.50806 | 0.612811 | 0.700355 |
| S1V_1 | -0.21086 | 0.33699 | -0.62572 | 0.533281 | 0.700355 |
| S1B_1 | -0.30719 | 0.299772 | -1.02475 | 0.308572 | 0.617144 |
| S1G_1 | 0.227409 | 0.220223 | 1.032627 | 0.30489 | 0.617144 |
| S1Y_1 | 0.321011 | 0.170474 | 1.883054 | 0.063328 | 0.506621 |
| S1R_1 | 0.176703 | 0.495644 | 0.356512 | 0.722396 | 0.722396 |
| H1 | 512.7198 | 338.9966 | 1.512463 | 0.134357 | 0.537427 |
| B2 | -27.5928 | 35.83644 | -0.76997 | 0.443588 | 0.700355 |
| **Belly** |  |  |  |  |  |
| S1U_1 | -0.40621 | 0.257122 | -1.57983 | 0.118091 | 0.188946 |
| S1V_1 | -0.48383 | 0.281868 | -1.71651 | 0.089939 | 0.188946 |
| S1B_1 | -0.40435 | 0.247487 | -1.63382 | 0.106226 | 0.188946 |
| S1G_1 | 0.155004 | 0.183459 | 0.844894 | 0.40069 | 0.457931 |
| S1Y_1 | 0.271413 | 0.137448 | 1.97466 | 0.051757 | 0.188946 |
| S1R_1 | 0.561869 | 0.427795 | 1.313406 | 0.192801 | 0.257068 |
| H1 | 689.5984 | 254.2256 | 2.712545 | 0.008172 | 0.065375 |
| B2 | -19.428 | 42.95695 | -0.45227 | 0.652301 | 0.652301 |

Supplementary table 3. Results of a PGLS (now with lambda optimized using ML) relating NDVI to chroma (S1U-R) S1U (300-400 nm), S1V (300-415 nm), S1B (400-510 nm), S1G (510-605 nm), S1Y (550-625 nm), S1R (605-700 nm) and hue (H1) for female colour.

|  | Estimate | Std. Error | t-val. | p-val. | p-val. cor. |
| --- | --- | --- | --- | --- | --- |
| **Crown** |  |  |  |  |  |
| S1U_1 | -0.08178 | 0.29665 | -0.27569 | 0.783595 | 0.945412 |
| S1V_1 | -0.09522 | 0.321337 | -0.29634 | 0.76785 | 0.945412 |
| S1B_1 | 0.049894 | 0.16402 | 0.304194 | 0.761882 | 0.945412 |
| S1G_1 | -0.00903 | 0.131462 | -0.06872 | 0.945412 | 0.945412 |
| S1Y_1 | -0.05437 | 0.089979 | -0.6042 | 0.547665 | 0.945412 |
| S1R_1 | -0.0504 | 0.400357 | -0.12589 | 0.900178 | 0.945412 |
| H1 | 42.43187 | 35.85114 | 1.183557 | 0.240592 | 0.945412 |
| B2 | 42.43187 | 35.85114 | 1.183557 | 0.240592 | 0.945412 |
| **Mantle** |  |  |  |  |  |
| S1U_1 | -0.40221 | 0.322417 | -1.2475 | 0.216373 | 0.432746 |
| S1V_1 | -0.46241 | 0.353905 | -1.30659 | 0.195629 | 0.432746 |
| S1B_1 | -0.44163 | 0.179813 | -2.45605 | **0.016532** | 0.132255 |
| S1G_1 | -0.06767 | 0.099759 | -0.67835 | 0.499784 | 0.666379 |
| S1Y_1 | 0.093661 | 0.084804 | 1.10444 | 0.273184 | 0.437095 |
| S1R_1 | 0.853486 | 0.525827 | 1.623133 | 0.109056 | 0.432746 |
| H1 | -71.8518 | 245.7177 | -0.29242 | 0.770834 | 0.823888 |
| B2 | 4.897708 | 21.92511 | 0.223384 | 0.823888 | 0.823888 |
| **Throat** |  |  |  |  |  |
| S1U_1 | -0.30765 | 0.295262 | -1.04197 | 0.301011 | 0.790811 |
| S1V_1 | -0.33706 | 0.329364 | -1.02336 | 0.309659 | 0.790811 |
| S1B_1 | 0.037385 | 0.209775 | 0.178214 | 0.85907 | 0.875833 |
| S1G_1 | -0.02066 | 0.131743 | -0.15683 | 0.875833 | 0.875833 |
| S1Y_1 | 0.082774 | 0.096799 | 0.855113 | 0.395406 | 0.790811 |
| S1R_1 | 0.262241 | 0.490475 | 0.534667 | 0.594574 | 0.792766 |
| H1 | -236.798 | 408.4013 | -0.57982 | 0.5639 | 0.792766 |
| B2 | 52.55838 | 38.41768 | 1.368078 | 0.175663 | 0.790811 |
| **Breast band 1** | |  |  |  |  |
| S1U_1 | -0.2122 | 0.257785 | -0.82317 | 0.413209 | 0.577639 |
| S1V_1 | -0.23624 | 0.27267 | -0.86639 | 0.389236 | 0.577639 |
| S1B_1 | -0.11377 | 0.153508 | -0.74116 | 0.461075 | 0.577639 |
| S1G_1 | 0.102791 | 0.120415 | 0.853646 | 0.396213 | 0.577639 |
| S1Y_1 | 0.091463 | 0.081356 | 1.124243 | 0.26475 | 0.577639 |
| S1R_1 | 0.196352 | 0.350972 | 0.559452 | 0.577639 | 0.577639 |
| H1 | 48.72397 | 84.46527 | 0.576852 | 0.56589 | 0.577639 |
| B2 | 94.61482 | 32.7955 | 2.884994 | 0.005199 | **0.04159** |
| **Breast band 2** | |  |  |  |  |
| S1U_1 | -0.31953 | 0.260723 | -1.22556 | 0.224471 | 0.299295 |
| S1V_1 | -0.34475 | 0.272087 | -1.26706 | 0.209333 | 0.299295 |
| S1B_1 | 0.044393 | 0.162896 | 0.272524 | 0.786022 | 0.786022 |
| S1G_1 | 0.136145 | 0.103707 | 1.31279 | 0.193542 | 0.299295 |
| S1Y_1 | 0.133973 | 0.081466 | 1.644524 | 0.104552 | 0.278805 |
| S1R_1 | 0.234698 | 0.284618 | 0.824609 | 0.412395 | 0.471308 |
| H1 | 147.2321 | 83.60835 | 1.760974 | 0.08261 | 0.278805 |
| B2 | 84.61187 | 42.91804 | 1.971475 | 0.052623 | 0.278805 |
| **Belly** |  |  |  |  |  |
| S1U_1 | -0.28151 | 0.236102 | -1.19233 | 0.237215 | 0.387143 |
| S1V_1 | -0.29628 | 0.240979 | -1.22947 | 0.22307 | 0.387143 |
| S1B_1 | -0.0024 | 0.19354 | -0.01241 | 0.990136 | 0.990136 |
| S1G_1 | 0.081256 | 0.106667 | 0.761773 | 0.448792 | 0.512905 |
| S1Y_1 | 0.104483 | 0.088528 | 1.180223 | 0.241964 | 0.387143 |
| S1R_1 | 0.189332 | 0.214707 | 0.881816 | 0.380938 | 0.507917 |
| H1 | 131.2612 | 86.18094 | 1.523089 | 0.132307 | 0.387143 |
| B2 | 80.0838 | 50.88361 | 1.573863 | 0.120094 | 0.387143 |
